# Global research landscape on nanotechnology in acute lung injury: a bibliometric analysis

**DOI:** 10.3389/fdgth.2025.1472753

**Published:** 2025-03-04

**Authors:** Jian Zhang, Shasha Jiang, Jipeng Jiang, Yang Liu

**Affiliations:** ^1^ School of Medicine, Nankai University, Tianjin, China; ^2^ Department of Thoracic Surgery, The First Medical Center of Chinese PLA General Hospital, Beijing, China; ^3^ Postgraduate School, Medical School of Chinese PLA, Beijing, China

**Keywords:** bibliometric analysis, nanotechnology, acute lung injury, drug delivery, research trend

## Abstract

**Background:**

Acute lung injury is a common respiratory emergency that seriously affects the life, health and quality of life of patients, especially after the global COVID-19 pneumonia. The application of nanotechnology in acute lung injury is promising. In response to the knowledge explosion resulting from rapid publication growth, we applied bibliometric analysis to explore the research profile and thematic trends in the field.

**Methods:**

Articles and reviews related to nanotechnology in acute lung injury from 2004 to 2023 were searched. Java-based Citespace, VOSviewer, and R software-based Bibiometrix were used to systematically evaluate publications by spatiotemporal distribution, author distribution, subject categories, topic distribution, references, and keywords.

**Results:**

A total of 1,347 publications were included. The number of papers related to nanotechnology in acute lung injury has grown exponentially over the past 20 years. China was the most productive country out of all 53 countries, followed by the United States. The Chinese Academy of Sciences was the most productive institution with 76 papers. *PARTICLE AND FIBRE TOXICOLOGY* was the most productive journal. The top five high-frequency keywords were inflammation, oxidative stress, toxicity, *in vitro*, respiratory-distress-syndrome. And the top five emerging keywords were delivery, covid-19, extracellular vesicles, therapy, sars-cov-2. Drug delivery are the focus of current research. Two emerging research areas represented the development trends: novel nanocarriers with higher efficiency and lower biotoxicity, and the other is research related to impact of nanomaterials in the progression of acute lung injury.

**Conclusion:**

The field of nanotechnology in acute lung injury has been in a period of rapid development in the last three years. Delivery,targeted delivery and exosm have been the focus of current research in this field. Two emerging research areas represented the development trends:novel nanocarriers with higher efficiency and lower biotoxicity such as extracellular vesicles, exosomes and solid lipid nanoparticles, and the other is research related to impact of nanomaterials in the progression of acute lung injury.

## Introduction

Acute lung injury (ALI) and acute respiratory distress syndrome (ARDS) are serious respiratory diseases (1). They present as acute and widespread pulmonary inflammation, impaired gas exchange, and hypoxemia, usually due to pulmonary or systemic factors, including pneumonia, aspiration trauma, and chest trauma (1–4). According to early studies, the mortality rate for ARDS was once as high as 50–60 percent (2). In recent years, studies have shown that, owing to improvements in mechanical ventilation strategies, this rate has fallen to between 35 and 46 percent (4). However, treatment of ALI and ARDS is primarily supportive, to maintain oxygenation and reduce lung damage (5–8). This leads to the possibility that patients who survive may experience a permanent decline in lung function, including restrictive or obstructive pulmonary hypoplasia (9–11). Particularly after the global COVID-19 pneumonia infection, a proportion of surviving patients still suffer from some degree of sclerosis and fibrosis of the lung tissue, which severely affects the quality of life (12–15). Therefore, early recognition and treatment were essential to improve prognosis in acute lung injury and ARDS. Nowadays, new therapeutic approaches were constantly being explored, especially stem cell therapy (16–20) and specific drug treatments (21–25).

Nanotechnology is a rapidly growing field. It can diagnose, treat, and prevent diseases by manipulating substances on the nanoscale (26–28). In the field of medicine, nanotechnology has an extremely wide range of applications including but not limited to drug delivery (29–31), diagnosis (32–34), imaging (35–37), therapy (28, 38–41), organizational engineering (27, 42), as well as infection control (43–46) and prevention (26, 47, 48). Presently, significant research on designing nanotechnology has been carried out and has made some research progress in the field of acute lung injury (31, 49–53). It is believed that shortly, nanomedicine can become an indispensable treatment in the field of acute lung injury.

Bibliometrics is a discipline that studies the quantitative process and law of scientific literature, which uses mathematics, statistics, and other methods to quantitatively analyze the production, distribution, dissemination, and use of scientific literature as a means of revealing the dynamics and law of scientific and technological development (54–56). In this study, we provide a comprehensive bibliometric analysis of the application of Nanotechnology in the field of acute lung injury from January 2004 to December 2023, providing a scientific performance of countries, institutions, authors, journals, and references, presenting collaborative networks and research trends from a global perspective.

## Materials and methods

### Data source and search strategy

Science Citation Index Expanded (SCI Expanded) of the Web of Science Core Compendium (WoSCC) was chosen as the data source. The search strategy was as follows: Topic = (nanoparticle* OR nanomedicines* OR nano* OR nanometer* OR nanomaterial* OR nanotechnology* OR nanoparticulate* OR nanocrystalline material* OR nanocomposites* OR nanoaggregates* OR nanocrystal* OR nano particle* OR nanocarrier* OR nanotherapeutic*) AND topic = ((ALI) OR (ARDS) OR (acute lung injury) OR (acute lung disease) OR (acute respiratory distress syndrome) OR (respiratory distress syndrome)) AND language = (English) AND publication year = (2004–2023). The author conducted a literature search and downloaded data in plain text or UTF-8 format. In addition, we have excluded specific types of publications and only include original articles and reviews. The literature and data search were completed on March 10th, 2024, to avoid bias caused by database updates.

### Data analysis and visualization

We summarized the bibliometric indicators of publications, including authors, affiliated institutions, countries, number of articles, journals, keywords, references, and collaborations. Citation, G-index, and H-index were used to evaluate the academic impact of authors or journals. Journal categories (Q1, Q2, Q3, and Q4) and impact factors (IF) were excerpted from the 2023 Journal Citation Report.

We conducted a comprehensive analysis using the Java-based Citespace (version 6.3.R1), the Java program VOS Viewer (version 1.6.18), the R software-based Bibiometrix (version 3.0), and the integrated online analysis platform (https://bibliometric.com/). Citespace (version 6.3.R1) was utilized to visualize and demonstrate the structure, patterns, and dissemination of scientific knowledge. The nodes in the maps represented institutions, while the connections between nodes represented collaborations. The gauge of the lines indicated the magnitude of the relationship, and the color denoted the year of initial occurrence.

Knowledge networks of nanotechnology in acute lung injury were assessed by using co-citation analysis, co-occurrence analysis, co-authorship analysis, citation burst detection, collaborative networks, and biplot overlap of journals of journals.

### Ethics statement

This study is primarily literature-based and quantitatively analysed using mathematical and statistical methods, and does not involve human participants, human specimens or tissue, vertebrate animals or cephalopods, vertebrate embryos or tissues, field research. The primary data for this study were sourced from previously published studies, all of which had already obtained ethical approval from their respective ethics committees. Consequently, no additional ethical approval was deemed necessary for the current study. All literature searches were conducted in Web of Science.

## Results

### General information about bibliometric analysis

**Figure 1:**
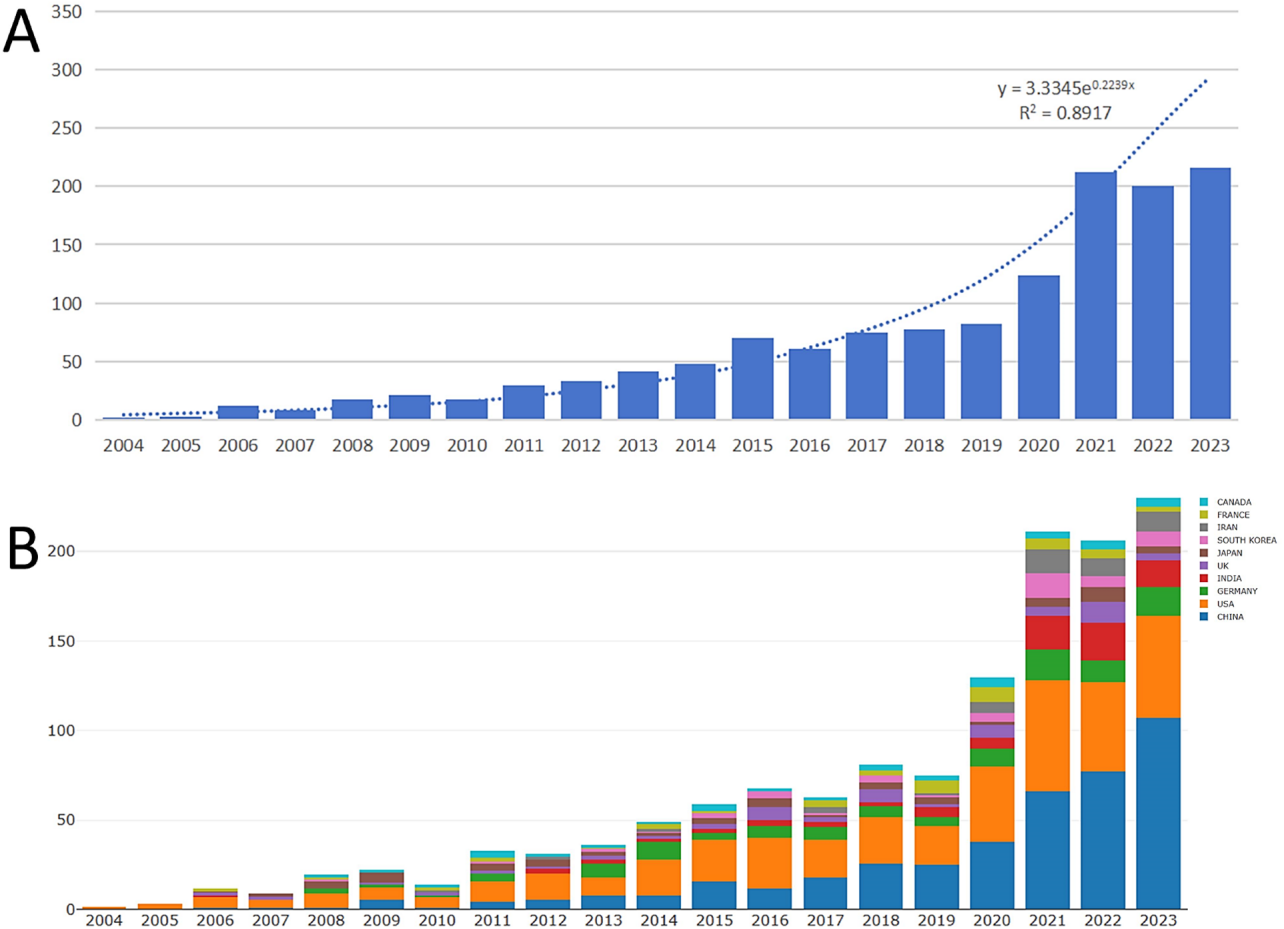
Annual publication trends in the world **(A)** and in the top ten most productive countries **(B).**

Table 1 describes the basic information of our bibliometric analysis. In the past 20 years, 1,347 publications on the application of nanotechnology in acute lung injury have been published in 524 journals. These publications were written by 8,520 authors, including 1,114 original papers and 233 reviews, with 72,712 references cited. The average number of citations per document per year was 34.31. As shown in Figure 1A, the exponential growth in the number of publications in recent years indicates potential development in this field. The last three years alone accounted for 46.6% (628/1,347) of the publications.

**Table 1:** Main information about bibliometric analysis.

Description	Results
Main information about data	
Timespan	2,004:2,023
Sources (Journals, Books, etc.)	524
Documents	1,347
Annual Growth Rate %	12.74
Document Average Age	5.31
Average citations per doc	34.31
References	72,712
Document contents	
Keywords Plus (ID)	3,959
Author's Keywords (DE)	3,575
Authors	
Authors	8,520
Authors of single-authored docs	18
Authors collaboration	
Single-authored docs	19
Co-Authors per Doc	8.44
International co-authorships %	30.36
Document types	
Article	1,114
Review	233

### Publication performances: countries

We evaluated the performance of these countries in publications based on the nationality of the corresponding authors. Fifty-three countries have published papers on this topic. As shown in Table 2, China was the country with the highest productivity (*n* = 388, 28.8%), followed by the United States (*n* = 324, 24.1%). The top ten countries with the highest productivity include one from North America, one from South America, five from Asia, and three from Europe. The number of publications in these countries has exploded, with China performing outstandingly in the past three years (Figure 1B). The United States has the highest number of citations [*n* = 16,324, average article citation (AAC) = 50.40], followed by China (*n* = 9,617, AAC=24.80) and France (*n* = 2,130, AAC = 73.40). Mexico has the highest citation count of 127.00.

**Table 2:** Top 20 productive countries and citations per country.

Sort by NP	Country	Articles (%)	SCP	MCP(%)	Sort by total citations	Country	Total citations	Average article citations
1st	CHINA	388 (28.8)	320	68 (17.5)	1st	USA	16,324	50.40
2nd	USA	324 (24.1)	247	77 (23.8)	2nd	CHINA	9,617	24.80
3rd	GERMANY	61 (4.5)	34	27 (44.3)	3rd	FRANCE	2,130	73.40
4th	INDIA	61 (4.5)	41	20 (32.8)	4th	GERMANY	2,021	33.10
5th	JAPAN	49 (3.6)	43	6 (12.2)	5th	JAPAN	2,005	40.90
6th	IRAN	40 (3.0)	28	12 (30.0)	6th	UNITED KINGDOM	1,214	50.60
7th	KOREA	37 (2.7)	29	8 (21.6)	7th	INDIA	1,154	18.90
8th	FRANCE	29 (2.2)	18	11 (37.9)	8th	KOREA	1,061	28.70
9th	BRAZIL	27 (2.0)	20	7 (25.9)	9th	BRAZIL	898	33.30
10th	ITALY	25 (1.9)	11	14 (56.0)	10th	ITALY	861	34.40
11th	CANADA	24 (1.8)	13	11 (45.8)	11th	DENMARK	770	32.10
12th	DENMARK	24 (1.8)	10	14 (58.3)	12th	MEXICO	762	127.00
13th	UNITED KINGDOM	24 (1.8)	9	15 (62.5)	13th	SWEDEN	732	43.10
14th	AUSTRALIA	22 (1.6)	8	14 (63.6)	14th	CANADA	644	26.80
15th	EGYPT	19 (1.4)	13	6 (31.6)	15th	SWITZERLAND	618	34.30
16th	SWITZERLAND	18 (1.3)	8	10 (55.6)	16th	IRAN	595	14.90
17th	SWEDEN	17 (1.3)	11	6 (35.3)	17th	SPAIN	573	38.20
18th	SPAIN	15 (1.1)	8	7 (46.7)	18th	IRELAND	572	95.30
19th	SAUDI ARABIA	14 (1.0)	3	11 (78.6)	19th	AUSTRALIA	421	19.10
20th	AUSTRIA	12 (0.9)	3	9 (75)	20th	AUSTRIA	378	31.50

NP, number of publications; MCP, multiple countries publications (inter-country collaboration); SCP, single country publications (intra-country collaboration).

**Figure 2:**
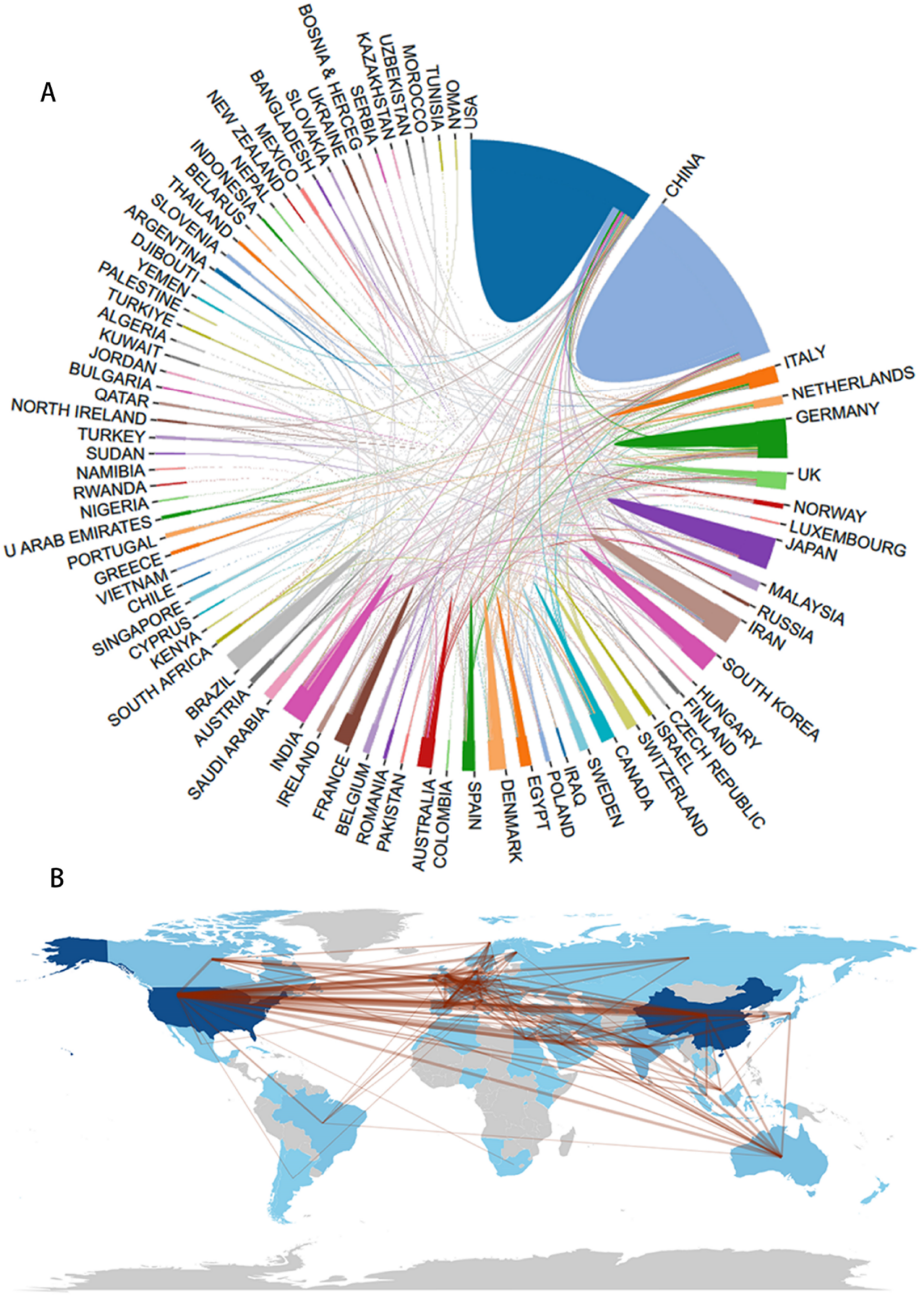
The country distribution **(A)** and international collaboration **(B)** of publications in the field of nanotechnology in acute lung injury.

We have built a global collaborative network for nanotechnology in acute lung injury. Figure 2A shows that although there was cooperation among many countries, the number of collaborative publications was still relatively low. We mapped our cooperation to a global map and found that the cooperation areas were mainly concentrated in North America, Europe, East Asia, and Oceania. The collaboration was primarily focused on the United States-China (Figure 2B).

### Publication performances: institutions

**Figure 3:**
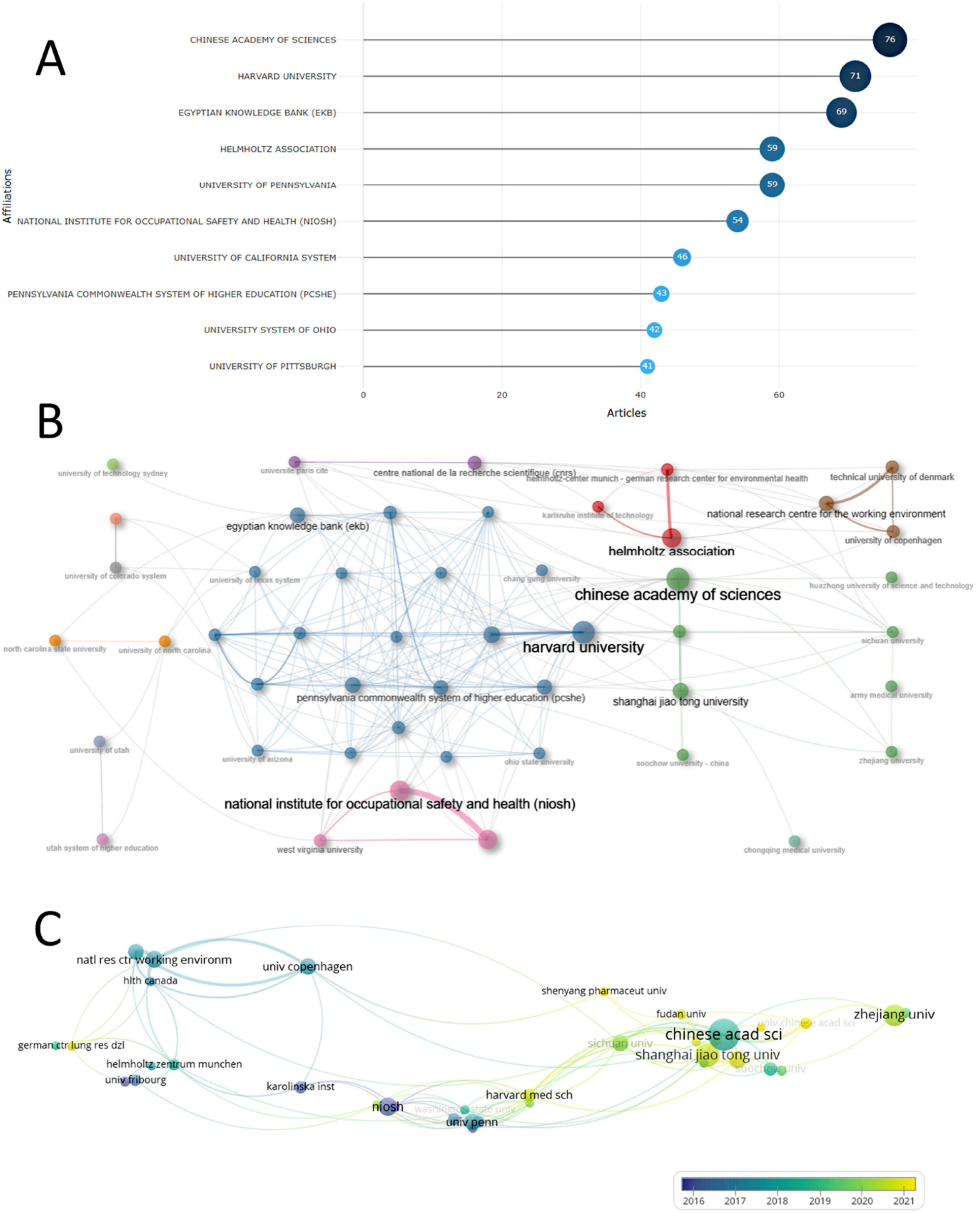
Publication performance of the institution: the top ten most productive institutions **(A)**; inter-institutional collaboration network **(B)**; and co-authorship analysis of institutions **(C).**

CHINESE ACADEMY OF SCIENCES was the most productive institution with 76 publications. Ranked second through fifth were HARVARD UNIVERSITY (*n* = 71), EGYPTIAN KNOWLEDGE BANK (EKB, *n* = 69), HELMHOLTZ ASSOCIATION (*n* = 59), and UNIVERSITY OF PENNSYLVANIA (*n* = 59). Seven of the top ten institutions were from the United States, one from China, one from Egypt, and one from Germany, demonstrating the significant influence of North America in this field (Figure 3A).

We conducted a co-authorship analysis of institutions with at least five publications (Figure 3C). The National Research Centre for the Working Environment had the highest total link strength (TLS) at 44, the second through fifth places were the Technical University of Denmark (*n* = 41), CHINESE ACADEMY OF SCIENCES (*n* = 41), University of Copenhagen (*n* = 34), and Shanghai Jiao Tong University (*n* = 26). Based on further indications from the collaborative network, we did not find an obvious core institution for the cluster, suggesting that inter-institutional collaboration was not close (Figure 3B).

### Publication performances: authors

**Figure 4:**
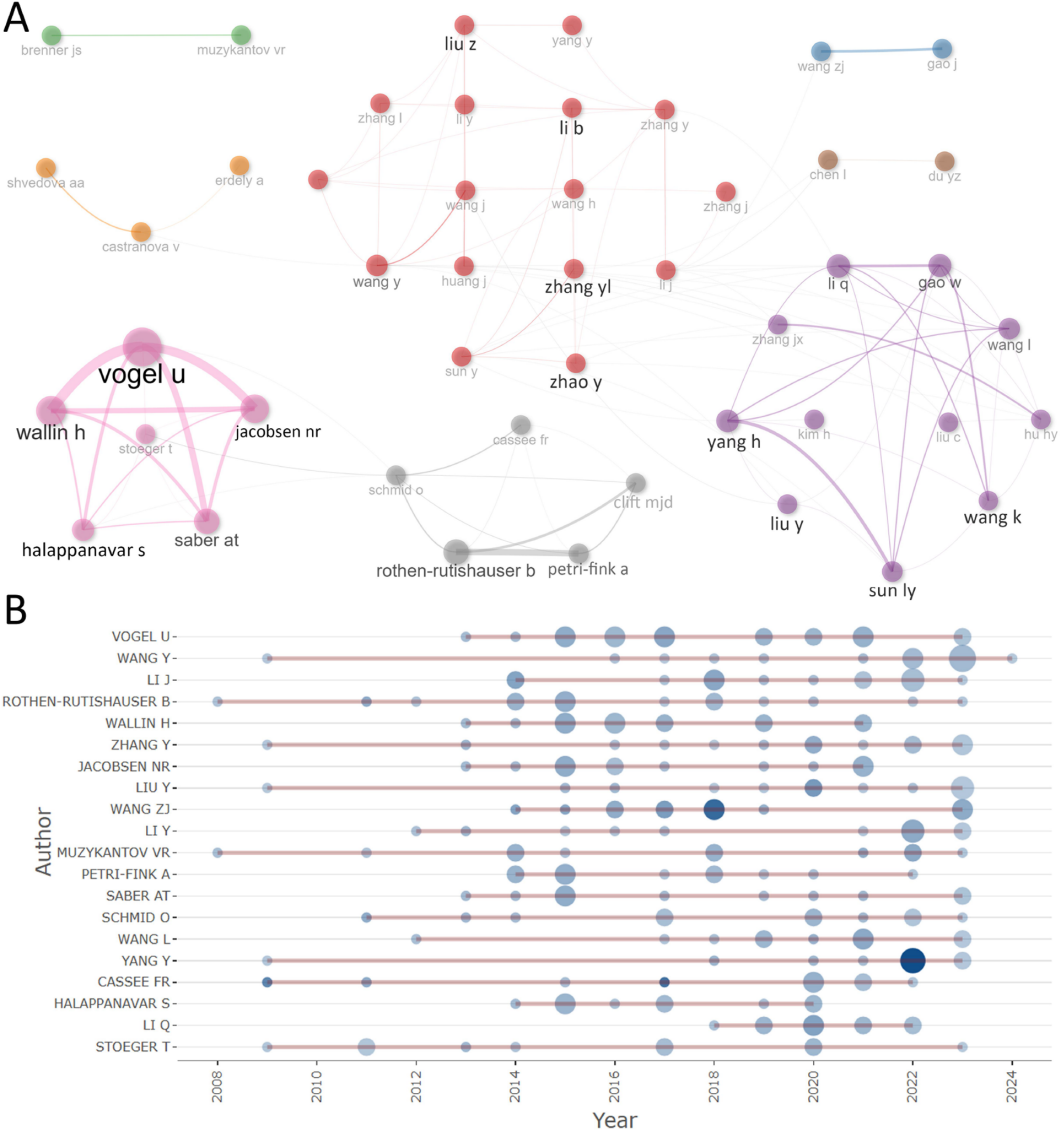
Publication performance of the authors: the inter-author collaboration network **(A)**; and top authors’ production over time **(B).**

The top ten most cited authors are shown in Table 3 and the most prolific authors are in Table 4. Curiously, it's hard to find a clear consistency between the number of publications and the total citations. DONALDSON K was the most cited author (TC = 1,760, NP = 4). REED KL, WARHEIT DB, and WEBB TR rank second in total citations (TC = 1,553, NP = 2). VOGEL U was the most prolific author (TC = 755, NP = 20). WANG Y ranks second in productivity (TC = 159, NP = 16). This proves that four papers researched by DONALDSON K were of high academic standard and reference value. Meanwhile, REED KL, WARHEIT DB, and WEBB TR might be co-authors of the same two high-level publications. As shown in Figure 4A, collaborative network analysis revealed eight author clusters, with Vogel U and Rothen-Rutishauser B located at the center of their respective clusters. We also analyzed the changes in the works of top authors over time (Figure 4B). Thirteen of the 20 authors have been deeply involved in this field for over a decade. Notably, most of the authors have experienced a scholarly explosion after 2014, with a staggering number of publications and citations.

**Table 3:** Top Ten most cited authors.

Authors	TC	H-index	G-index	NP	Country
DONALDSON K	1,760	4	4	4	UK
REED KL	1,553	2	2	2	USA
WARHEIT DB	1,553	2	2	2	USA
WEBB TR	1,553	2	2	2	USA
CASTRANOVA V	1,443	9	9	9	USA
SHVEDOVA AA	1,377	8	8	8	USA
CASSEE FR	1,344	9	10	10	Netherlands
LI B	1,323	5	7	7	China
WANG ZJ	1,227	12	13	13	USA
LAURENCE BR	1,164	1	1	1	USA

NP, number of publications; TC, total citations.

**Table 4:** Top Ten most prolific authors.

Authors	TC	H-index	G-index	NP	Country
VOGEL U	755	15	20	20	Denmark
WANG Y	159	7	12	16	China
ROTHEN-RUTISHAUSER B	737	13	15	15	Netherlands
LI J	494	8	15	15	China
WALLIN H	639	13	14	14	Denmark
ZHANG Y	345	9	14	14	China
WANG ZJ	1,227	12	13	13	USA
JACOBSEN NR	560	11	13	13	Denmark
LIU Y	353	7	13	13	China
LI Y	336	8	12	12	China

### Highly contributive journals

**Figure 5:**
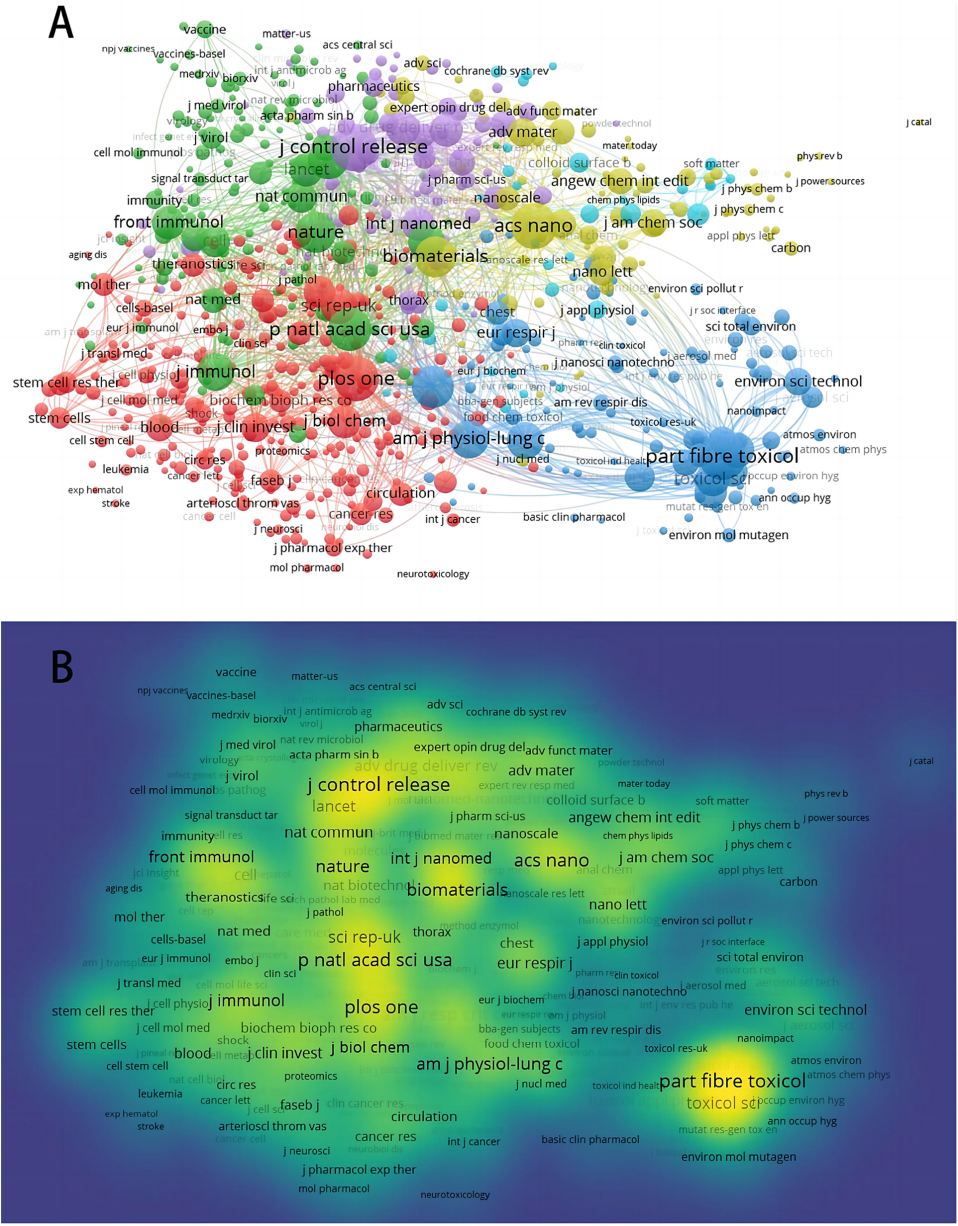
Publication performance of the journals: co-citation network **(A)**; and density visualization **(B).**

The most productive journal published on nanotechnology in acute lung injury was *PARTICLE AND FIBRE TOXICOLOGY*. It published 41 related publications and received 2,433 citations. The *JOURNAL OF CONTROLLED RELEASE* (*n* = 27, citations = 1,115) and *ACS NANO* (*n* = 26, citations = 1,514) were second and third. Three of the top ten most productive journals belonged to JCR Q1 and four to JCR-Q2 (Table 5). This proves that most of the journals on this topic have a high academic impact. Setting the minimum number of citations to 20 in the journal co-citation network (Figure 5), *JOURNAL OF CONTROLLED RELEASE* had the highest total link strength (TLS) of 124,338. The second through fifth places were *PLOS ONE* (TLS = 99,759), *ACS NANO* (TLS = 96,112), *PROCEEDINGS OF THE NATIONAL ACADEMY OF SCIENCES OF THE UNITED STATES OF AMERICA* (TLS = 95,500) and *BIOMATERIALS* (TLS = 83,959). The visualization of the density of co-citation networks revealed that *ACS NANO, PARTICLE AND FIBRE TOXICOLOGY, PROCEEDINGS OF THE NATIONAL ACADEMY OF SCIENCES OF THE UNITED STATES OF AMERICA, JOURNAL OF CONTROLLED RELEASE* and *PLOS ONE* in the core position.

**Table 5:** The top ten most productive journals.

Sort by number of articles	Relevant sources	Articles	TC	H-Index	JCR	IF (2023)
1st	PARTICLE AND FIBRE TOXICOLOGY	41	2,433	83	Q1	10
2nd	JOURNAL OF CONTROLLED RELEASE	27	1,115	237	Q1	10.8
3rd	ACS NANO	26	1,514	310	Q1	17.1
4th	INTERNATIONAL JOURNAL OF NANOMEDICINE	25	655	100	Q2	8
5th	INTERNATIONAL JOURNAL OF MOLECULAR SCIENCES	22	423	114	Q2	5.6
6th	NANOTOXICOLOGY	22	586	72	Q3	5
7th	FRONTIERS IN PHARMACOLOGY	21	268	62	Q2	5.6
8th	PLOS ONE	21	942	268	Q3	3.7
9th	TOXICOLOGICAL SCIENCES	20	2,362	164	Q3	3.8
10th	ACS APPLIED MATERIALS & INTERFACES	18	280	169	Q2	9.5

**Figure 6:**
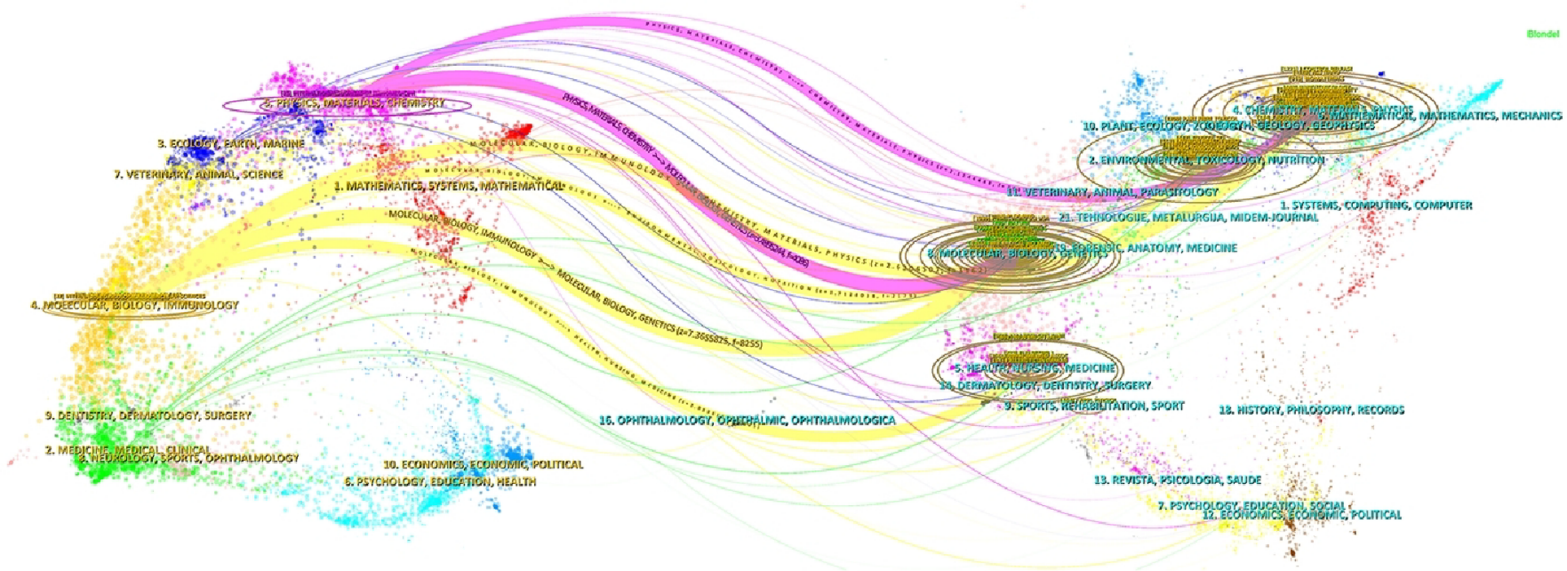
A dual-map overlap of the research journals.

Dual map overlay of journals shows the distribution of topics (Figure 6). The citing journals are on the left, and the cited journals are on the right. The labels represent the disciplines covered by the journals, and the colored path represented the citation relationship. We can see the most important six paths. The two purple citation paths indicate that research in Chemistry/Materials/Physics journals and Molecular/Biology/Genetics journals were frequently cited by Chemistry/Materials/Physics journals. The four yellow citation paths indicate that research in Chemistry/Materials/Physics journals, Molecular/Biology/Genetics journals, Environmental/Toxicology/Nutrition journals and Healthy/Nursing/Medicine journals were frequently cited by Molecular/Biology/Immunology journals.

### Highly contributive papers

**Figure 7:**
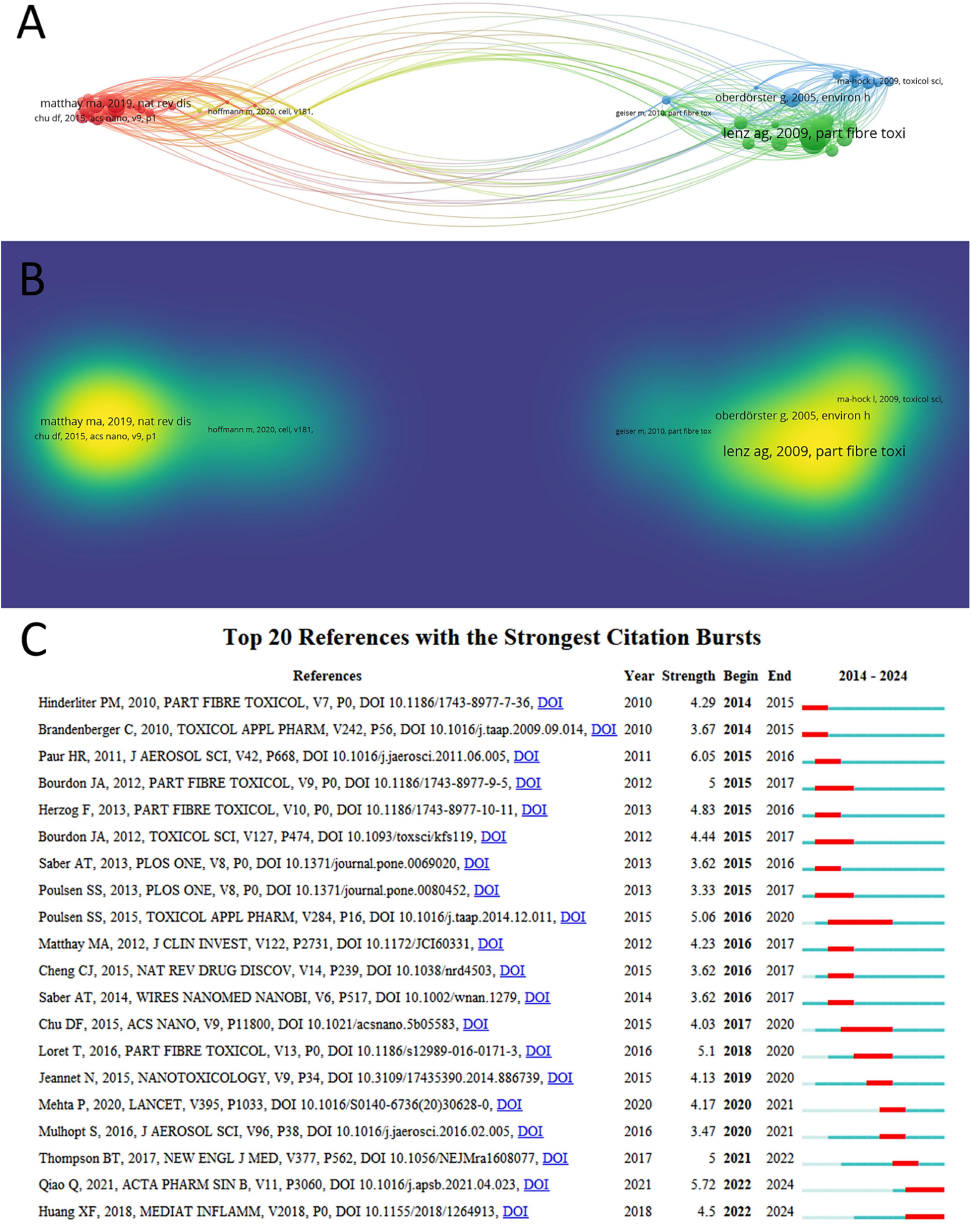
Highly contributive papers: a co-citation network **(A)** and density visualization **(B)** of references; and the top 20 references with the strongest citation bursts **(C).**

Table 6 lists the top ten most cited articles. These highly cited studies were published from 2004 to 2020, with 8 of them published after 2010. The most cited paper was written by WARHEIT DB (57) and published in 2004, which compared pulmonary toxicity of single-wall carbon nanotubes in rats. We constructed a co-citation reference network (Figures 7A,B). It consists of the four highest clusters of TLS publications written by LENZ AG(TLS = 257), Oberdörster G(TLS = 151), MATTHAY MA(TLS = 158), and HOFFMANN M(TLS = 51). Figure 7C shows the top 20 references with the strongest citation burst. The journal with the greatest contribution was *PART FIBRE TOXICOL*, with a total of 4 publications, followed by *TOXICOL APPL PHARM, J AEROSOL SCI*, and *PLOS ONE*, all with a total of 2 publications. The citation explosion occurred after 2014 and experienced rapid turnover.

**Table 6:** Top Ten articles related to nanotechnology in acute lung injury.

Articles	DOI	Year	Global citations	Local citations
WARHEIT DB, 2004, TOXICOL SCI	10.1093/toxsci/kfg228	2004	1,164	18
DONALDSON K, 2010, PART FIBRE TOXICOL	10.1186/1743-8977-7-5	2010	662	22
SHVEDOVA AA, 2008, AM J PHYSIOL-LUNG C	10.1152/ajplung.90287.2008	2008	511	15
PAUR HR, 2011, J AEROSOL SCI	10.1016/j.jaerosci.2011.06.005	2011	228	48
CHU DF, 2015, ACS NANO	10.1021/acsnano.5b05583	2015	189	26
UPADHYAY S, 2018, TOXICOL SCI	10.1093/toxsci/kfy053	2018	163	18
ZHAO H, 2018, NANOSCALE	10.1039/c8nr00838h	2018	160	12
KLEIN SG, 2013, PART FIBRE TOXICOL	10.1186/1743-8977-10-31	2013	147	18
RAO L, 2020, P NATL ACAD SCI USA	10.1073/pnas.2014352117	2020	146	11
GAO J, 2017, BIOMATERIALS	10.1016/j.biomaterials.2017.05.003	2017	134	14

### Keyword co-occurrence

**Figure 8:**
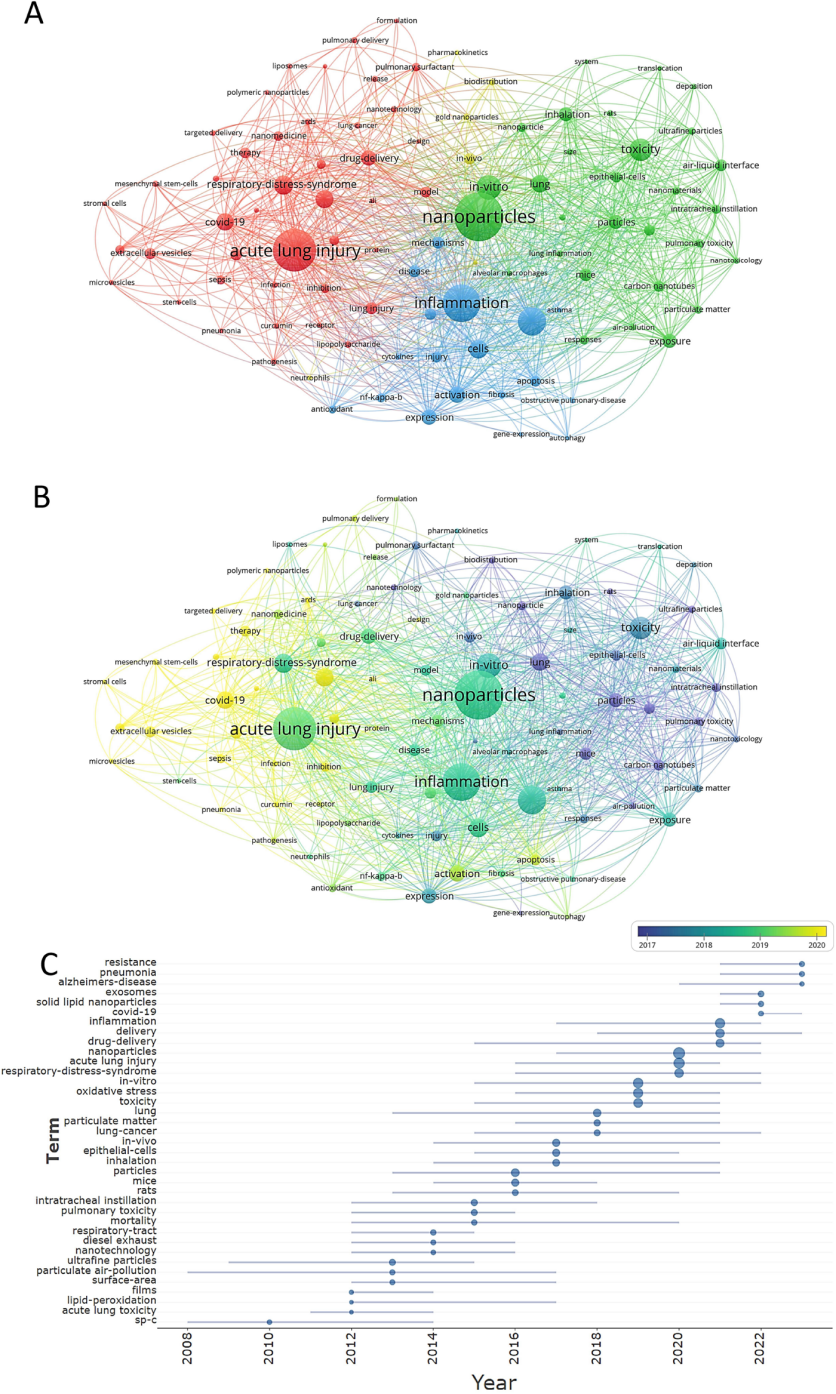
Cluster analysis **(A)**, temporal evolution **(B)**, and trends **(C)** of the keywords.

VOSviewer parameters were as follows: Method (Linlog/modularity) and a minimum number of occurrences of a keyword: 20. There were 6,892 keywords, with 95 keywords meeting the thresholds. For each of 95 keywords, the total strength of co-occurrence links with other keywords was calculated. Keywords with the greatest total link strength were selected. The keyword co-occurrence network graph (Figure 8A) displays that the thicker the connection between nodes, the higher the frequency of two keywords appearing together. Keywords formed four clusters, representing the four main research directions nanotechnology and acute lung injury.

The red cluster mainly includes acute lung injury, respiratory-distress-syndrome, delivery, covid-19, drug-delivery, lung injury, extracellular vesicles, therapy, nanomedicine, exosomes, drug delivery, model, sars-cov-2, inhibition, cancer, sepsis, pulmonary surfactant, lipopolysaccharide, ards, nanotechnology, mesenchymal stem-cells, pathogenesis, curcumin, pulmonary delivery, microvesicles, release, acute respiratory distress syndrome, infection, targeted delivery, liposomes, stromal cells, receptor, formulation, nanocarriers, polymeric nanoparticles, coronavirus, pneumonia, stem-cells, ali, protein, design, and lung-cancer.

The green cluster mainly contains nanoparticles, toxicity, *in vitro*, lung, exposure, particles, inhalation, mice, air-liquid interface, epithelial-cells, cytotoxicity, carbon nanotubes, intratracheal instillation, pulmonary toxicity, nanoparticle, responses, nanomaterials, ultrafine particles, particulate matter, nanotoxicology, air-pollution, lung inflammation, rats, silver nanoparticles, deposition, size, alveolar macrophages, translocation, and system.

The blue cluster mainly comprises inflammation, oxidative stress, cells, activation, expression, macrophages, mechanisms, injury, apoptosis, disease, nf-kappa-b, fibrosis, antioxidant, cytokines, asthma, autophagy, obstructive pulmonary-disease, and gene-expression.

The yellow cluster mainly consists of *in vivo*, biodistribution, gold nanoparticles, pulmonary inflammation, neutrophils, and pharmacokinetics.

In addition to nanoparticles (TLS = 2,357) and acute lung injury (TLS = 1,784), keywords with higher frequency in this study include inflammation (TLS = 1,808), oxidative stress (TLS = 1,364), toxicity (TLS = 1,040), *in vitro* (TLS = 1,030), respiratory-distress-syndrome (TLS = 674), activation (TLS = 634), covid-19 (TLS = 626), particles (TLS = 616), drug-delivery (TLS = 605), and inhalation (TLS = 601). Among these keywords, all of these TLS appeared more than 600 times, indicating that they were the focus of research.

The temporal evolution of keywords was shown in Figure 8B. The more yellow color indicates that the keyword was more emerging. The top ten high-frequency emerging keywords were delivery (TLS = 654), covid-19 (TLS = 626), extracellular vesicles (TLS = 410), therapy (TLS = 376), sars-cov-2 (TLS = 354), exosomes (TLS = 332), cancer (TLS = 291), sepsis (TLS = 285), inhibition (TLS = 281), and mesenchymal stem-cells (TLS = 218). Additionally, we conducted keyword trend analyses (Figure 8C). The research hotspots in the last three years were delivery/drug-delivery/inflammation (2021), exosomes/solid lipid nanoparticles/covid-19 (2022), resistance/pneumonia/alzheimers-disease (2023).

### Keyword burst detection

**Figure 9:**
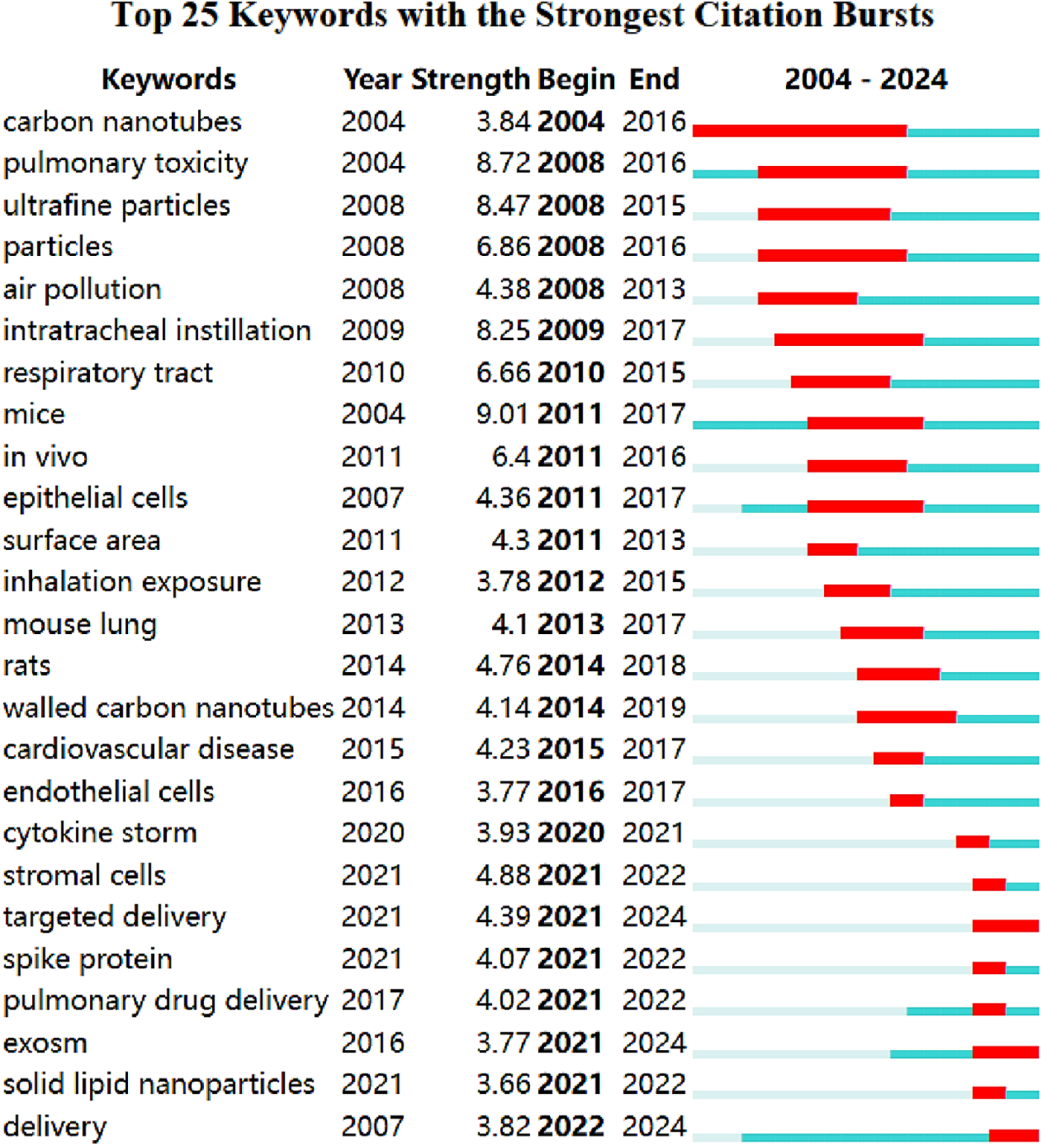
Burst detection of the keywords.

Combined with Keyword explosion detection, we further analyzed the popularity trend of keywords. Figure 9 intuitively shows the stage hotspots and development directions of nanotechnology and acute lung injury research from the time dimension. The blue line denotes the time axis while the red segment on the blue time axis demonstrates the burst detection, indicating the start year, end year, and burst duration. The top 25 keywords with the strongest citation bursts in nanotechnology and acute lung injury were carbon nanotubes (2004), pulmonary toxicity (2004), ultrafine particles (2008), particles (2008), air pollution (2008), intratracheal instillation (2009), respiratory tract (2010), mice (2011), *in vivo* (2011), epithelial cells (2011), surface area (2011), inhalation exposure (2012), mouse lung (2013), rats (2014), walled carbon nanotubes (2014), cardiovascular disease (2015), endothelial cells (2016), cytokine storm (2020), stromal cells (2021), targeted delivery (2021), spike protein (2021), pulmonary drug delivery (2021), exosm (2021), solid lipid nanoparticles (2021), and delivery (2021). Targeted delivery (2021–2024), exosm (2021–2024), and delivery (2021–2024) were the current research frontiers in nanotechnology and acute lung injury, and they were already in an explosive period.

## Conclusion

We provided an insight into the global knowledge landscape and trends in nanotechnology in acute lung injury through bibliometric analysis. China was a leader in this field. Delivery,targeted delivery and exosm have been the focus of current research in this field. Two emerging research areas represented the development trends:novel nanocarriers with higher efficiency and lower biotoxicity such as extracellular vesicles, exosomes and solid lipid nanoparticles, and the other is research related to impact of nanomaterials in the progression of acute lung injury. This report could serve as a reference and guide for more in-depth studies in the future.

## Discussion

This study systematically presents the global research overview and macro patterns of publications in the field of nanotechnology in acute lung injury by spatiotemporal distribution, author distribution, subject categories, topic distribution, references, and keywords. It was expected that this study will serve as a reference and guide for analyzing the current status of the field and for more in-depth research in the future.

Our findings show a significant increase over the past 20 years. Although the use of nanotechnology in acute lung injury is not new, we have been faced with a dramatic increase in the literature over the past three years (46.6%, 628/1,347). China (*n* = 388, 28.8%) was the most productive country out of all 53 countries, followed by the United States (*n* = 324, 24.1%). The top ten countries with the highest productivity include one from North America, one from South America, five from Asia, and three from Europe. The United States has the highest number of citations [*n* = 16,324, average article citation (AAC) = 50.40], followed by China (*n* = 9,617, AAC = 24.80) and France (*n* = 2,130, AAC = 73.40). Mexico has the highest citation count of 127.00. The global collaborative network for nanotechnology in acute lung injury showed that although there was cooperation among many countries, the number of collaborative publications was still relatively low. We mapped our cooperation to a global map and found that the cooperation areas were mainly concentrated in North America, Europe, East Asia, and Oceania. The collaboration was primarily focused on the United States-China. As a result, China and the United States are international leaders in this field and have developed a deep level of co-operation. It is easy to understand, as the development of high technology is closely related to economic power.

Furthermore, seven of the top ten institutions in terms of publications were from the United States, one from China, one from Egypt, and one from Germany, demonstrating the significant influence of North America in this field. However, the Chinese Academy of Sciences was the most published institution with 76 papers. This indicates that Chinese research institutions were also making important contributions in this field. It also shows that other Chinese research organisations have a greater potential for development. Deeper multi-agency co-operation is likely to be one of the main directions for the future.

Analysis of high-frequency keywords reflects the hotspots in a particular research field. We explored the evolution of research concepts and hot-spots in the field through keyword co-occurrence analysis and burst detection. Cluster analysis based on keywords, finally forming a cluster of four colors. Then, according to the analysis of the top 25 keywords with the strongest citation bursts, the research hotspots and frontiers in nanotechnology and acute lung injury were determined. The present study revealed that nanoparticle drug delivery was the most important and cutting-edge application of nanotechnology in acute lung injury, and it was foreseeable that a greater number of investigations would focus on this area in the future.

Nanomedicine is an interdisciplinary science that combines nanoscience, nano-engineering, and nanotechnology with life sciences (57–60). The field of nanomedicine aims to utilize the nature and physical properties of nanomaterials to diagnose and treat diseases at the molecular level (61). Until now, nanotechnology has been an extremely wide range of applications including but not limited to drug delivery (29–31), diagnosis (32–34), imaging (35–37), therapy (28, 38–41), organizational engineering (27, 42), as well as infection control (43–46) and prevention (26, 47, 48). Nanomaterials were a key part of nanomedicine. There were many types of nanomaterials, many of which can replicate the functions of some spherical biomolecules. For example, liposomes (62), different polymer nanostructures (63), protein structures (64), carbon dots (65), nanodiamonds (66), carbon nanotubes (67), graphene, and inorganic nanomaterials such as mesoporous silica (MSNP) (68), superparamagnetic iron oxide nanomaterials (SPIONs) (69), and quantum dots (QDs) (70). These nanomaterials have different applications in the field of medicine due to their different properties.

Meanwhile, researchers have continuously optimised the composition and structure of nanomaterials to obtain nanocarriers with higher drug delivery efficiency and lower biotoxicity. And the effect of nanomaterials in disease progression has also been intensively researched (31, 49–53). In the progression of ARDS, excessive production of reactive oxygen species (ROS) can lead to uncontrolled inflammatory responses. A 2023 study found that the functional nanomaterial Molybdenum nanodots (MNDs), with superior properties such as ultra-small size, good biocompatibility and excellent ROS scavenging ability, could protect lung tissue by inhibiting the activation of NOD-like receptor protein 3 (NLRP3) (52). The oxidative stress, inflammation, protein permeability, and histological severity of ALI mice were significantly improved by intratracheal administration (52). Cerium oxide nanoparticle (CNP) carriers, constructed in another study in 2021, can deliver unstable therapeutic drugs such as anti-inflammatory microRNA-146a locally to injured lungs via intratracheal administration without systemic absorption (49). CNP-miR146a improves lung biomechanics in acute lung injury after bleomycin exposure by altering leukocyte recruitment, reducing inflammation and oxidative stress, and reducing collagen deposition (49). Liposomes prepared using the thin-film aqueous method can bind to the epigenetic regulatory protein BRD4 to construct BRD4 siRNA and cationic lipid complexes (71). BRD4 siRNA liposomes (BRD4-SIRNA-LP) inhibit lung inflammatory cells, lipopolysaccharide (LPS) -induced neutrophil infiltration and mast cell aggregation, and LPS-induced cytokine storms and inflammatory signaling pathways, and improve lung compliance (71).

Carbon nanotubes are one of the most common nanomaterials. According to Figure 9, we discovered the use of carbon nanotubes in acute lung injury as early as 2004. Carbon nanotubes present a wide range of application prospects due to their unique structure and excellent mechanical, electrical and chemical properties. It has attracted great attention from many scientists in the fields of materials, physics, electronics, chemistry, etc., and has become the research frontier and hotspot in the field of international new materials (72). Furthermore,magnetic nanoparticles recently in few decades have proved as an effective advanced drug delivery system for its elevated magnetic responsiveness, biocompatibility, elevated targeted drug delivery effectiveness. In the presence of an external magnetic field, the drug can be easily targeted to the active site. Owing to their easy execution towards drug delivery application, extensive research has been carried out in this area (73).

In the wake of the COVID-19 pandemic, vaccination has emerged as the most effective method of disease prevention, and public confidence in vaccines depends on their safety and efficacy (74). Among them, the delivery of proteins and DNA/RNAs has become an important challenge (74, 75).The field of vaccine development is entering a new nano-era. In July 2020, the SARS-CoV-2 vaccine portfolio included 158 vaccine candidates (74, 75). Approximately 20 of these agents were in advanced stages of development, including mRNA-based vaccines, adenovirus-based vaccines, and pathogen-specific vaccines (74, 75). In the summer of 2020, experimental vaccines showing promising results in clinical trials were based on inactivated or attenuated live viruses, protein subunits, virus-like particles, viral vectors, and chemically synthesized nanoparticles (NPs, liposomes) delivered with DNA and mRNA (75). So far dozens of COVID-19 vaccines are in clinical trials or have passed clinical trials (76–80). And according to the keyword trend chart (Figure 8C),We find that COVID-19 becomes a popular keyword in 2022, which indicates that 2022 is an output year for COVID-19-related scientific research.

In addition to synthetic nanoparticles, bioderived nanoscale extracellular vesicles have also become a research hotspot (81–83). Compared with synthetic nanoparticles, extracellular vesicles (EVs) have good biocompatibility, low immunogenicity, natural cell targeting, and complex biomolecular loading capacity (84–86). Exosomes isolated from conditioned cultures of lentivirus-transfected mouse pulmonary microvascular endothelial cells (MPMVECs) were found to be useful for intratracheal injections for the treatment of lipopolysaccharide (LPS)-induced ALI in mice (87). Studies have found that inhalation of exosomes can improve pulmonary edema and inflammation, reduce the number of cells and protein levels in bronchoalveolar lavage fluid (BALF), and reduce the expression of pro-inflammatory cytokines such as IL-1 beta, TNF-α and IL-6 (87). In another study, neutrophil membrane-engineered Panax ginseng root-exosome (N-exo)-loaded microRNA (miRNA) 182-5p (N-exo-miRNA 182-5p) was found to have a favourable therapeutic effect on acute lung injury (ALI) in sepsis (88). The study found that N-exo-miRNA 182-5p significantly improved ALI by targeting and modulating the NOX4/Drp-1/NLRP3 signaling pathway *in vivo* and *in vitro* (88).

In addition, through the above studies, we found that in the treatment of lung diseases, aerosol inhalation has become an indispensable drug delivery route (49, 52, 87). Aerosol inhalation delivers drugs directly to the lungs and airways. Compared with the intravenous route, aerosol inhalation of the drug mainly acts locally, reducing the amount entering the systemic circulation, which may reduce systemic side effects (85–90). Thus, biogenic extracellular vesicle nebulized inhalation has great potential for the treatment of acute lung injury.

It should be noted that our study still has some limitations. First, although WoSCC is the most commonly used data source for bibliometric analysis. However, ignoring publications from other databases may lead to selection bias. Therefore, more rigorous analyses should be conducted in the future using more databases. Second, only SCI-Expanded articles and reviews in English were included in this study. Next, open-source journals have an impact on both citations and publication sit require a certain amount of time for an article to reach a high academic impact after publication. Finally, bibliometric analyses were usually conducted annually, and some recently published literature was not included in the assessment. Given the rapid growth of the field, more findings may emerge in future updated studies.

## Data Availability

The original contributions presented in the study are included in the article/Supplementary Material, further inquiries can be directed to the corresponding authors.
